# Primary cilia in retinal pigment epithelium development and diseases

**DOI:** 10.1111/jcmm.16882

**Published:** 2021-08-27

**Authors:** Chunjiao Sun, Jun Zhou, Xiaoqian Meng

**Affiliations:** ^1^ College of Life Sciences Shandong Provincial Key Laboratory of Animal Resistance Biology Institute of Biomedical Sciences Shandong Normal University Jinan China

**Keywords:** barrier, development, primary cilium, retinal disease, retinal pigment epithelium

## Abstract

Retinal pigment epithelium (RPE) is a highly polarized epithelial monolayer lying between the photoreceptor layer and the Bruch membrane. It is essential for vision through participating in many critical activities, including phagocytosis of photoreceptor outer segments, recycling the visual cycle‐related compounds, forming a barrier to control the transport of nutrients, ions, and water, and the removal of waste. Primary cilia are conservatively present in almost all the vertebrate cells and acts as a sensory organelle to control tissue development and homeostasis maintenance. Numerous studies reveal that abnormalities in RPE lead to various retinal diseases, such as age‐related macular degeneration and diabetic macular oedema, but the mechanism of primary cilia in these physiological and pathological activities remains to be elucidated. Herein, we summarize the functions of primary cilia in the RPE development and the mutations of ciliary genes identified in RPE‐related diseases. By highlighting the significance of primary cilia in regulating the physiological and pathological processes of RPE, we aim to provide novel insights for the treatment of RPE‐related retinal diseases.

## 1 INTRODUCTION

**FIGURE 1:**
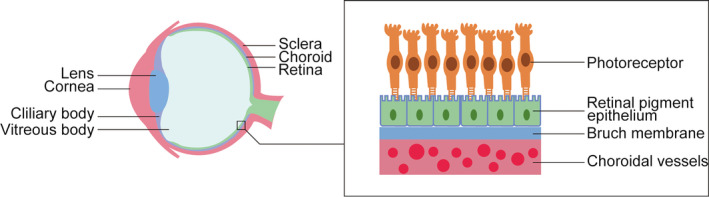
Close‐up of the eyeball. The cornea resides in front of the eyeball, and the choroid and retina lie in the inner part of the eyeball. The mature retinal pigment epithelial cells forma single layer (RPE) which lies between the Bruch membrane and the retina

Retinal pigment epithelium (RPE), derived from the embryonic neuroepithelium, is a highly polarized epithelial monolayer which lies between the photoreceptor layer and the Bruch membrane (Figure 1). In the early embryonic stage, the neuroepithelium produces an inner layer and an outer layer. The inner layer develops into the retinal nerve layer, and the outer layer develops into retinal pigment epithelium.^1^ RPE layer is essential for the homeostasis and visual function of the retina.^2^ As an important part of the blood‐retina barrier, RPE layer controls the transport of nutrients, ions, metabolic waste products, and water into and out of the retina.^3^, ^4^ In addition, RPE layer regulates retinal metabolism, gobbles up the outer segments of photoreceptor, and absorbs the stray light which is not captured by the photoreceptor.^5^

Primary cilia are microtubule‐based hair‐like structures present in almost all the vertebrate cells. They consist of basal body, transition zone, axoneme and ciliary membrane. Many receptors and signal molecules are enriched in ciliary membrane, enabling primary cilia as an antenna to sense and transduce extracellular clues, thus regulating many cellular activities, including cell proliferation and differentiation.^6^ Defects in primary cilia are associated with a range of diseases that have emerged as ‘ciliopathies’. Primary cilia are detected in cornea, trabecular network and retina. Interestingly, primary cilia are dynamically changed during the developmental and maturation process of RPE, and defective primary cilia of RPE layer are associated with retina‐related diseases, inferring the indispensable role for primary cilia in RPE.^7^ Herein, we review the alterations of primary cilia during the RPE development and the physiological and pathological roles of primary cilia in regulating RPE development and homeostasis, aiming to highlight the significance of primary cilia in RPE and provide therapeutic strategies for RPE‐related diseases.

## 2 PRIMARY CILIA AND RPE DEVELOPMENT

**FIGURE 2:**
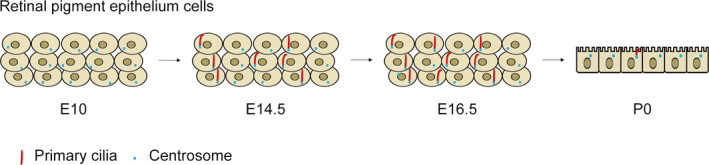
The primary cilia expression pattern during RPE development. During the RPE development in mice, primary cilia begin to assemble at E14.5, and the number of ciliated cells reach the highest at E16.5, at which the ciliary length is the longest. At P0 the RPE are fully mature, forming a single layer with cubic cells aligning in a neat row, and only a few cells have short primary cilia. The short red lines indicate primary cilia and the small blue dots represent the centrosomes

Primary ciliogenesis is a dynamic process oscillating during the cell cycle. Primary cilia are formed in G_0_ phase and resorbed as cells re‐enter the cell cycle. Similarly, primary cilia are changed dynamically during RPE development. During mice embryonic development, primary cilia are detectable in RPE layer at E14.5, and reach the highest to 70% at E16.5, and resorb at E18.5 with a decreased ciliary length. In the RPE layer of postnatal mouse, primary cilia can be observed only in a little of cells^8^ (Figure 2). The narrow expression window of primary cilia during the development of the RPE suggests that primary cilia may play a crucial role in RPE and their activity must be tightly controlled.

A recent study revealed that RPE is crucial for photoreceptor development and function, and primary cilia is required for complete maturation of RPE probably through WNT pathway.^9^ Defects in primary ciliogenesis lead to RPE immaturation and consequently photoreceptor degeneration.^9^ Several ciliary genes in ciliopathies are found to be responsible for RPE development and retinal functions. For example, Bardet‐Biedl syndrome 8(BBS8), an important member of BBSome complex, is critical for the biogenesis of the ciliary membrane and ciliary protein trafficking. *BBS8* gene mutations are associated with retinitis pigmentosa and early vision loss through affecting the development of outer segments in photoreceptor neurons.^10^ Centrosomal protein 290 (CEP290) is essential for the assembly of ciliary transition zone, and its mutations are frequently found in Leber congenital amaurosis (LCA), an autosomal recessive childhood blindness disorder.^11^

Retinal pigment epithelium 65(RPE65), an isomerase in the visual cycle, is highly expressed in the RPE. Light that reaches the RPE activates the visual pigment and converts the all‐trans retinol to all‐trans retinol, which in turn is oxidized by RPE65 to 11‐cis retinol.^12^ Retinitis pigmentosa 1(RP1), a homolog of RPE65, is located at the ciliary axeneme and its mutation is associated with retinitis pigmentosa (RP).^13^ It is worth exploring whether RPE65 is involved in primary ciliogenes is and RPE development.^14^ In addition, primary cilia play an important role in the development of RPE; however, the ciliated cells remarkably decrease in mature RPE, it is thus interesting to investigate the physiological role of primary cilia in mature RPE.

## 3 PRIMARY CILIA AND RPE‐RELATED DISEASES

**FIGURE 3:**
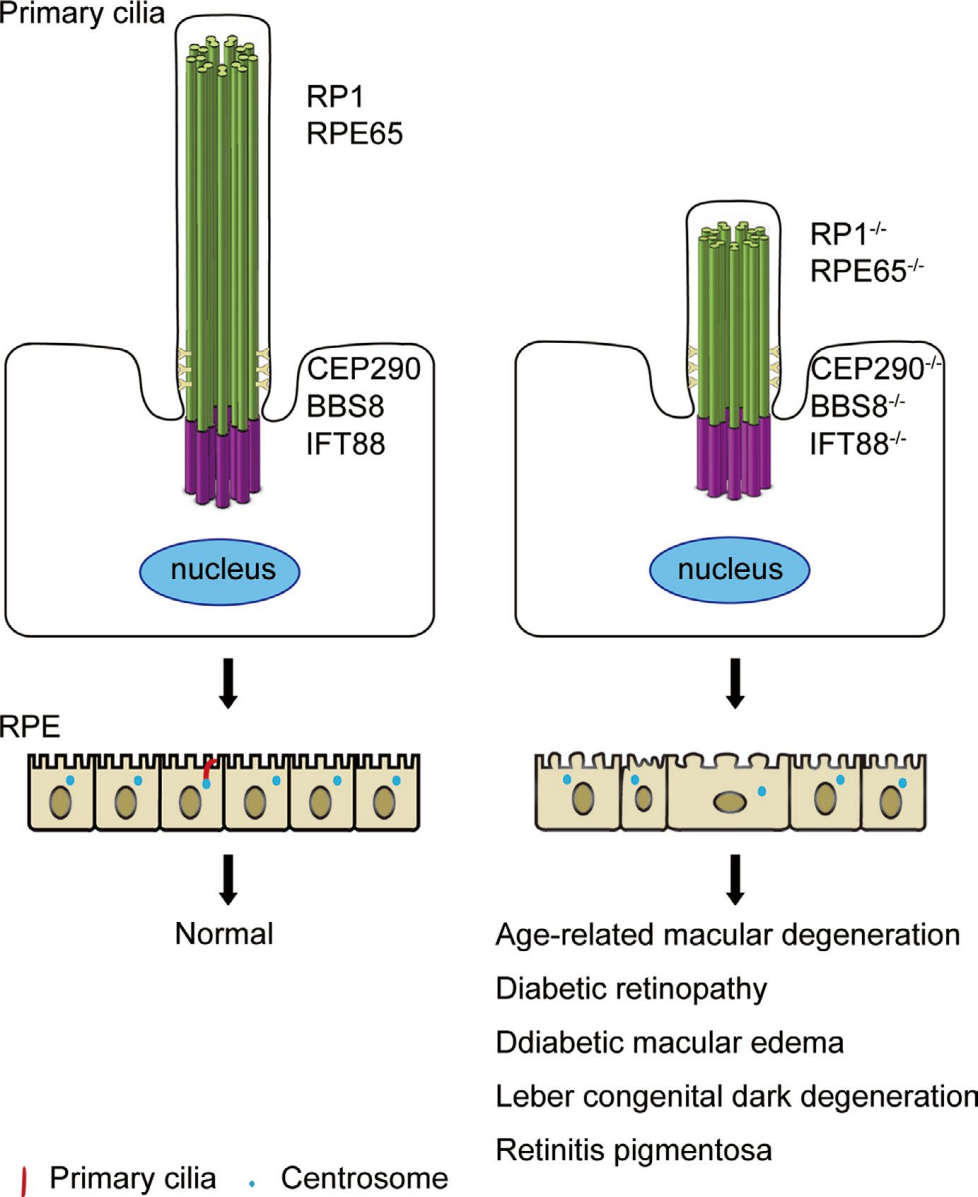
Abnormal primary cilia lead to RPE dysfunction and related diseases. Primary cilia regulate RPE maturation and homeostasis through WNT pathways. Mutations of the ciliary genes, such as RP1, RPE65, CEP290, BBS8, and IFT88 are often found in retinal diseases. Loss‐of‐function of these ciliary proteins may lead to defects in primary ciliogenesis, which in turn results in immature RPE, thereby contributing to retinal diseases, including diabetic retinopathy, diabetic macular edema, and age‐related macular degeneration. The short red lines indicate primary cilia and the small blue dots represent the centrosomes

Abnormal structure and function of RPE leads to impaired blood‐retina barrier, which affects cell diffusion, active transport and metabolism, thereby contributing to a large number of retinopathies, such as diabetic retinopathy (DR) and diabetic macular oedema (DME)^15^ (Figure 3). For example, the abnormal transport function of RPE leads to the accumulation of nutrients in the RPE, thereby generating insoluble extracellular aggregates, which in turn results in the degeneration of the RPE and photoreceptors, eventually causing visual impairment and retinal diseases.^16^ In addition, LCA and retinitis pigmentosa (RP) may be resulted from the structural and functional abnormalities of the RPE. Numerous studies have demonstrated that primary cilia and ciliary proteins are widely involved in these retinal diseases.

As shown in Figure 3, mutations of ciliary genes in RPE has been found to be associated with retinal diseases, such as DR, DME and LCA. LCA is an early‐onset retinal disease that causes blindness in children. The main clinical feature of LCA is retinal malnutrition, which leads to different degrees of visual impairment in patients. To date, at least 20 LCA mutated genes have been identified, including RPE65, CEP290 and CRB1.^17^ CEP290 intron mutation is the most common cause of LCA, which leads to splicing errors and premature termination.^18^ This causes defects in primary ciliogenesis and RPE transport.^19^ The frameshift mutation of pre‐mRNA processing factor 31 (PRPF31) suppressed primary ciliogenesis, resulted in an incorrect connection between the RPE and photoreceptor and impaired barrier function^20^, ^21^ (Figure 3). Although the association of ciliary gene mutations with ciliary defects and retinal diseases have been observed, the causal relationship and the underlying mechanisms of how primary cilia lead to RPE‐related diseases remain to be characterized.

## 4 TREATMENT OF RPE‐RELATED DISEASES

Gene therapy has been clinically used to correct the mutated genes for retinal diseases,^22^ of which the most used delivery vector is recombinant adeno‐associated virus (AAV).^23^ AAV‐based gene therapy targeting RPE cells has shown promising therapeutic prospects for RP patients with PRPF31 mutations. Inducing pluripotent stem cells (iPSCs) harbouring PRPF31 mutations differentiation into RPE cells is able to recapitulate PRPF31 mutation‐caused pathological phenotypes. AAV‐based PRPF31 gene therapy can restore the defects in phagocytosis and ciliary formation of RPE, showing promising therapeutic prospect for RP patients.^24^, ^25^ By sub‐retinal injection of AAV2‐RPE65 into LCA patients for dual‐allele replacement, visual impairment in RPE can be restored, and the clinical trials are currently ongoing.^12^ The abnormal splicing of CEP290 can be effectively blocked by antisense oligonucleotides in LCA patients.^19^ In addition, CRISPR/Cas9 technology has also been successfully used to correct CEP290 mutations in LCA patients.^26^

In addition, stem‐cell therapy has shown the potential for the treatment of retinal diseases.^27^ RPE patch composed of RPE monolayers differentiated from human embryonic stem cells (hESCs) was implanted into the sub‐retinal space of patients with AMD. After a period of time, the biological microscope and optical coherence tomography showed that the RPE patch could improve the vision of AMD patient.^28^ Notably, the conditioned medium used for hESC induction contained PDGF‐AA, TGF‐α and IGFBP‐2, which is conducive to primary ciliogenesis and RPE functions.^29^ Moreover, mature retinal organoid generated by co‐culture of human epidermal keratinocytes and human pluripotent stem cells contains primary cilia and is able to replace defective RPE and photoreceptors for the treatment of AMD patients.^30^

## 5 CONCLUSIONS AND PERSPECTIVES

Emerging studies reveal that functional RPE is essential for photoreceptor development and function. Primary cilia as an important signalling hub are critical for REP maturation and homeostasis maintenance. Although numerous mutations of ciliary genes in RPE‐related diseases have been identified, the significance of primary cilia in RPE has not attract enough attentions, and few studies have delved into the mechanism by which these mutations cause retinal diseases. In addition, the exact mechanisms of primary cilia in regulating RPE development and activity remain unclear. Particularly, a few of primary cilia are remained in mature RPE, suggesting primary cilia may play a differentiated role during RPE development. However, the role of the remnant primary cilia in mature RPE remains elusive, are they indispensable or acting as ‘stem cells’? Considering the promising potential of retinal organoid, it is thus attempting to investigate the roles and regulatory mechanisms of primary cilia in the activities of mature RPE, which may provide novel strategies for the treatment of retinal diseases.

## CONFLICT OF INTEREST

The authors confirm that there are no conflicts of interest.

## AUTHOR CONTRIBUTIONS

**Chunjiao Sun:** Software (lead); writing‐original draft (lead). **Jun Zhou:** Funding acquisition (equal); supervision (equal); visualization (equal); writing‐review & editing (equal). **Xiaoqian Meng:** Conceptualization (lead); funding acquisition (equal); supervision (lead); writing‐review & editing (lead).

## Data Availability

Data sharing is not applicable to this article as no new data were created or analyzed in this study.

## References

[1] Saini JS , Temple S , Stern JH . Human retinal pigment epithelium stem cell (RPESC). Adv Exp Med Biol. 2016;854:557‐562. 26427459 10.1007/978-3-319-17121-0_74

[2] Shang P , Stepicheva NA , Hose S , Zigler JS Jr , Sinha D . Primary cell cultures from the mouse retinal pigment epithelium. J Vis Exp. 2018;133:56997. 10.3791/56997 PMC5931770 29608155

[3] Ramsay E , Hagström M , Vellonen KS , et al. Role of retinal pigment epithelium permeability in drug transfer between posterior eye segment and systemic blood circulation. Eur J Pharm Biopharm. 2019;143:18‐23. 31419586 10.1016/j.ejpb.2019.08.008

[4] Strauss O . The retinal pigment epithelium in visual function. Physiol Rev. 2005;85(3):845‐881. 15987797 10.1152/physrev.00021.2004

[5] Lehmann GL , Benedicto I , Philp NJ , Rodriguez‐Boulan E . Plasma membrane protein polarity and trafficking in RPE cells: past, present and future. Exp Eye Res. 2014;126:5‐15. 25152359 10.1016/j.exer.2014.04.021 PMC4502961

[6] Álvarez‐Satta M , Moreno‐Cugnon L , Matheu A . Primary cilium and brain aging: role in neural stem cells, neurodegenerative diseases and glioblastoma. Ageing Res Rev. 2019;52:53‐63. 31004829 10.1016/j.arr.2019.04.004

[7] Pala R , Alomari N , Nauli SM . Primary cilium‐dependent signaling mechanisms. Int J Mol Sci. 2017;18:2272. 10.3390/ijms18112272 PMC5713242 29143784

[8] Patnaik SR , Kretschmer V , Brücker L , et al. Bardet‐Biedl Syndrome proteins regulate cilia disassembly during tissue maturation. Cell Mol Life Sci. 2019;76(4):757‐775. 30446775 10.1007/s00018-018-2966-x PMC11105770

[9] May‐Simera HL , Wan Q , Jha BS , et al. Primary cilium‐mediated retinal pigment epithelium maturation is disrupted in ciliopathy patient cells. Cell Rep. 2018;22(1):189‐205. 29298421 10.1016/j.celrep.2017.12.038 PMC6166245

[10] Dilan TL , Singh RK , Saravanan T , et al. Bardet‐Biedl syndrome‐8 (BBS8) protein is crucial for the development of outer segments in photoreceptor neurons. Hum Mol Genet. 2018;27(2):283‐294. 29126234 10.1093/hmg/ddx399 PMC5886228

[11] Zhang W , Li L , Su Q , Gao G , Khanna H . Gene therapy using a miniCEP290 fragment delays photoreceptor degeneration in a mouse model of Leber congenital amaurosis. Hum Gene Ther. 2018;29(1):42‐50. 28679290 10.1089/hum.2017.049 PMC5770090

[12] Miraldi Utz V , Coussa RG , Antaki F , Traboulsi EI . Gene therapy for RPE65‐related retinal disease. Ophthalmic Genet. 2018;39(6):671‐677. 30335549 10.1080/13816810.2018.1533027

[13] Yamashita T , Liu J , Gao J , et al. Essential and synergistic roles of RP1 and RP1L1 in rod photoreceptor axoneme and retinitis pigmentosa. J Neurosci. 2009;29(31):9748‐9760. 19657028 10.1523/JNEUROSCI.5854-08.2009 PMC2748320

[14] Dez AS , Efstratiou A . PCR typing of *Corynebacterium* *diphtheriae* by random amplification of polymorphic DNA. J Med Microbiol. 1999;48(4):335‐340. 10509474 10.1099/00222615-48-4-335

[15] Chawan‐Saad J , Wu M , Wu A , Wu L . Corticosteroids for diabetic macular edema. Taiwan J Ophthalmol. 2019;9(4):233‐242. 31942428 10.4103/tjo.tjo_68_19 PMC6947754

[16] Xia T , Rizzolo LJ . Effects of diabetic retinopathy on the barrier functions of the retinal pigment epithelium. Vision Res. 2017;139:72‐81. 28347688 10.1016/j.visres.2017.02.006

[17] Li G , Gao G , Wang P , et al. Generation and characterization of induced pluripotent stem cells and retinal organoids from a Leber's congenital amaurosis patient with novel RPE65 mutations. Front Mol Neurosci. 2019;12:212. 31572124 10.3389/fnmol.2019.00212 PMC6749091

[18] Drivas TG , Holzbaur EL , Bennett J . Disruption of CEP290 microtubule/membrane‐binding domains causes retinal degeneration. J Clin Invest. 2013;123(10):4525‐4539. 24051377 10.1172/JCI69448 PMC3784542

[19] Parfitt DA , Lane A , Ramsden CM , et al. Identification and correction of mechanisms underlying inherited blindness in human iPSC‐derived optic cups. Cell Stem Cell. 2016;18(6):769‐781. 27151457 10.1016/j.stem.2016.03.021 PMC4899423

[20] Nazlamova L , Thomas NS , Cheung MK , et al. A CRISPR and high‐content imaging assay compliant with ACMG/AMP guidelines for clinical variant interpretation in ciliopathies. Hum Genet. 2021;140(4):593‐607. 33095315 10.1007/s00439-020-02228-1 PMC7981318

[21] Buskin A , Zhu L , Chichagova V , et al. Disrupted alternative splicing for genes implicated in splicing and ciliogenesis causes PRPF31 retinitis pigmentosa. Nat Commun. 2018;9(1):4234. 30315276 10.1038/s41467-018-06448-y PMC6185938

[22] Collins M , Thrasher A . Gene therapy: progress and predictions. Proc Biol Sci. 1821;2015(282):20143003. 10.1098/rspb.2014.3003 PMC4707739 26702034

[23] Robert MA , Chahal PS , Audy A , Kamen A , Gilbert R , Gaillet B . Manufacturing of recombinant adeno‐associated viruses using mammalian expression platforms. Biotechnol J. 2017;12:1600193. 10.1002/biot.201600193 28177193

[24] Brydon EM , Bronstein R , Buskin A , Lako M , Pierce EA , Fernandez‐Godino R . AAV‐mediated gene augmentation therapy restores critical functions in mutant PRPF31(+/‐) iPSC‐derived RPE cells. Mol Ther Methods Clin Dev. 2019;15:392‐402. 31890732 10.1016/j.omtm.2019.10.014 PMC6909184

[25] Wheway G , Schmidts M , Mans DA , et al. An siRNA‐based functional genomics screen for the identification of regulators of ciliogenesis and ciliopathy genes. Nat Cell Biol. 2015;17(8):1074‐1087. 26167768 10.1038/ncb3201 PMC4536769

[26] Ruan GX , Barry E , Yu D , Lukason M , Cheng SH , Scaria A . CRISPR/Cas9‐mediated genome editing as a therapeutic approach for Leber congenital amaurosis 10. Mol Ther. 2017;25(2):331‐341. 28109959 10.1016/j.ymthe.2016.12.006 PMC5368591

[27] Alió Del Barrio JL , Alió JL . Cellular therapy of the corneal stroma: a new type of corneal surgery for keratoconus and corneal dystrophies. Eye Vis (Lond). 2018;5:28. 30410944 10.1186/s40662-018-0122-1 PMC6211455

[28] da Cruz L , Fynes K , Georgiadis O , et al. Phase 1 clinical study of an embryonic stem cell‐derived retinal pigment epithelium patch in age‐related macular degeneration. Nat Biotechnol. 2018;36(4):328‐337. 29553577 10.1038/nbt.4114

[29] Gu J , Wang Y , Cui Z , et al. The construction of retinal pigment epithelium sheets with enhanced characteristics and cilium assembly using iPS conditioned medium and small incision lenticule extraction derived lenticules. Acta Biomater. 2019;92:115‐131. 31075513 10.1016/j.actbio.2019.05.017

[30] Shrestha R , Wen YT , Ding DC , Tsai RK . Aberrant hiPSCs‐derived from human keratinocytes differentiates into 3D retinal organoids that acquire mature photoreceptors. Cells. 2019;8(1):36. 10.3390/cells8010036 PMC6356277 30634512

